# White-tailed deer milk promotes rapid SARS-CoV-2 RNA degradation through synergistic ionic and enzymatic mechanisms

**DOI:** 10.1016/j.isci.2026.118136

**Published:** 2026-11-06

**Authors:** Lorena P. Tobar, Wraylyn Smith, Hannah A. Raso, Katherine L. Brown, Alessandro Ceci, Matthew G. Urbano, Clinton Roby, Dzenis Mahmutovic, Andrew P. Biesemier, Daniel G.S. Capelluto, Ignacio Aiello, Dominic S. Raso, Carla V. Finkielstein

**Affiliations:** 1 Fralin Biomedical Research Institute at VTC, Roanoke, VA, USA; 2 Department of Dairy Science, Virginia Tech, Blacksburg, VA, USA; 3 Pathology Consultants of Central Virginia, Lynchburg, VA, USA; 4 Department of Pathology, University of Virginia, Charlottesville, VA, USA; 5 Department of Biological Sciences, Virginia Tech, Blacksburg, VA, USA; 6 Fralin Life Sciences Institute, Blacksburg, VA, USA

**Keywords:** SARS-CoV-2, white-tailed deer, COVID-19, milk, wildlife immunity, neutralizing antibodies, maternal-offspring transmission, lactoperoxidase

## Abstract

White-tailed deer (*Odocoileus virginianus*, WTD), the most extensively documented SARS-CoV-2 wildlife host in North America, undergo seasonal lactation that constitutes a direct yet uninvestigated pathway for viral exposure at the maternal-offspring interface. Here, we report the first detection of SARS-CoV-2-specific neutralizing antibodies in wildlife milk and demonstrate that WTD milk rapidly degrades viral RNA from three SARS-CoV-2 variants (lineages A, B.1.1.7, and BA.1.1.529) within 10 min at physiological temperatures, while human milk permits viral RNA persistence. Mechanistic interrogation revealed 5- to 20-fold elevated mineral concentrations, 3- to 5-fold enhanced protease activity, and substantially higher lactoperoxidase levels in WTD milk. Synergistic interactions among these ionic, proteolytic, and enzymatic factors drive variant-specific RNA degradation. Functional reconstitution of human milk with lactoperoxidase and a WTD-matched ionic formulation recapitulated the antiviral phenotype, establishing the mechanistic sufficiency of this biochemical axis. These findings identify species-specific milk biochemistry as a previously unrecognized determinant of viral fate during lactation.

## Highlights

• Wildlife lactation constitutes an immune barrier against SARS-CoV-2
• Deer milk actively clears viral RNA within minutes at body temperature
• Convergent biochemical defenses ensure robust antiviral protection
• Deer-derived factors confer antiviral capacity to human milk *in vitro*

## Graphical abstract

**Figure:**
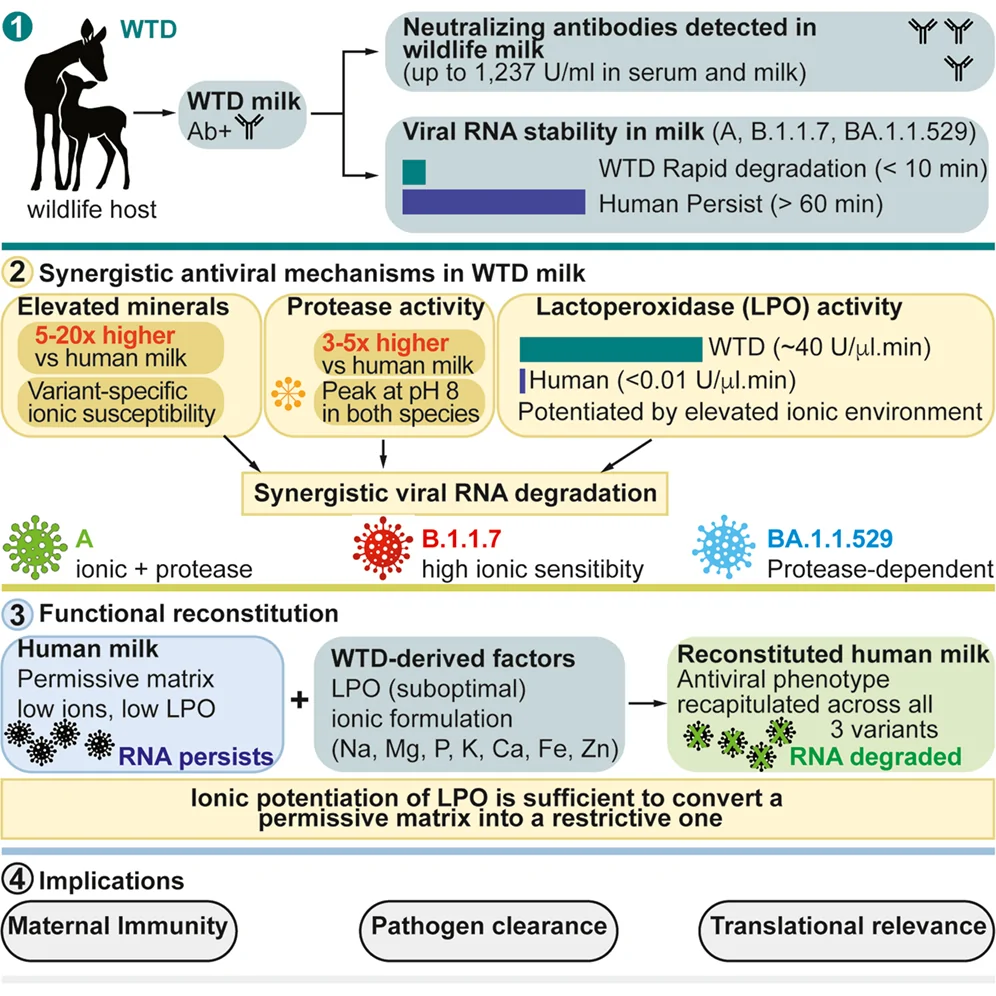

## Teaser

Biological sciences; Medical Microbiology; Viral microbiology; Proteomics

## Introduction

The severe acute respiratory syndrome coronavirus 2 (SARS-CoV-2) pandemic has demonstrated extensive cross-species transmission potential, with natural infections documented in over 30 mammalian species through both human-to-animal and animal-to-animal transmission pathways.^1^^,^^2^ Among wildlife species, white-tailed deer (*Odocoileus virginianus*, hereafter WTD) have emerged as the most extensively documented wildlife host since initial detection in late 2021, with widespread infection reported across North America.^3^^,^^4^^,^^5^ SARS-CoV-2 RNA and seroprevalence studies report infection rates of 15–40% across surveyed populations, with evidence of sustained viral circulation and heterogeneous spatial distribution of infection.^3^^,^^4^^,^^5^^,^^6^ The epidemiological significance of this wildlife reservoir is amplified by the estimated 30–34 million WTD population across North America, the frequent human-wildlife interfaces that occur through hunting, farming, and suburban contact,^7^ and the documented capacity for deer-to-deer transmission to sustain viral circulation independently of continued human spillover.^5^

The establishment of susceptible wildlife host populations creates ongoing risks for viral persistence, evolution, and potential reverse zoonotic transmission.^8^ Reverse zoonotic transmission from this host is documented rather than hypothetical, since a divergent SARS-CoV-2 lineage that emerged in WTD in Ontario, Canada, was subsequently identified in an epidemiologically linked human case.^9^ Characterization of SARS-CoV-2 in these hosts has nonetheless focused predominantly on seroprevalence and viral detection in respiratory secretions. Biological matrices that influence within-host viral fate, particularly milk, remain biologically uncharacterized among wildlife species.

Lactation is a period of intense, repeated contact, and fluid exchange between mother and offspring, and milk constitutes a complex biofluid whose functions extend well beyond nutrition.^10^ Across mammals, this matrix can both harbor viruses and shape immune and microbial ecology, making it directly relevant to understanding within-host pathogen dynamics.^11^ In the case of SARS-CoV-2, viral RNA is rarely detected in human milk, infectious virus is absent, and no case of infant infection has been linked to breastfeeding.^12^ Conversely, neutralizing antibodies, mainly IgA, are consistently present in human milk from infected and vaccinated mothers, suggesting passive immune protection for nursing infants.^13^ Despite these findings, SARS-CoV-2 dynamics in milk from infected wildlife populations remain uninvestigated, representing a substantial gap in our mechanistic understanding of pathogen fate at the maternal-offspring interface.

This gap is particularly significant because mammalian milks are not interchangeable biological matrices. Across species, milks exhibit vast, phylogenetically and ecologically linked differences in macronutrients, micronutrients, bioactive compounds, pH, ionic strength, and antimicrobial factors,^14^ which could substantially influence viral stability and immune factor activity in ways that cannot be predicted from human milk studies alone. Among cervids, milk demonstrates unusually high protein, fat, calcium, and distinctive fatty acid profiles compared to domestic dairy ruminants.^15^ These contrasts underscore that milk-based pathogen dynamics are simultaneously pathogen-specific and species-specific, as exemplified by the markedly different stability of avian influenza A (H5N1) in bovine versus other ruminant milk matrices,^16^ and the prolonged infectivity and documented transmission of Rift Valley fever virus through raw ruminant milk.^17^^,^^18^ Each pathogen-host-matrix combination therefore demands independent empirical characterization.

In this study, mammary gland tissue from the harvested does was examined by histology and immunohistochemistry for SARS-CoV-2 nucleocapsid. Rare SARS-CoV-2-positive histiocytes were present within mammary lobules, with no evidence of replication in mammary epithelial cells, identifying a potential source of cell-associated viral particles in milk. We therefore undertook a systematic, mechanistically grounded comparison of SARS-CoV-2 antibody response and viral RNA fate in WTD versus human milk. Rather than treating milk merely as a potential transmission vector, we approached it as a biochemically complex microenvironment whose species-specific properties could either facilitate or actively oppose viral persistence. Our results demonstrate the presence of neutralizing antibodies and distinct physicochemical properties in WTD milk that promote rapid viral RNA degradation, providing the first characterization of SARS-CoV-2 dynamics in wildlife milk and revealing synergistic ionic, proteolytic, and enzymatic-dependent mechanisms that collectively constitute a biochemical barrier to viral persistence.

## Results

### Specimen collection and regional SARS-CoV-2 variant surveillance

Milk, serum, and mammary gland tissue were collected from eight lactating WTD does harvested in Amherst, Bedford, and Nelson counties, central Virginia, USA, during the 2022–2024 hunting seasons (Figure S1). Human milk was obtained from two lactating volunteers (h.s.#1 and h.s.#2) sampled at multiple time points. SARS-CoV-2 variant distribution was established from 14,446 human swab samples collected in the same geographic area and time frame, of which 654 reverse-transcription - quantitative PCR (RT-qPCR)-positive specimens underwent whole-genome sequencing and Phylogenetic Assignment of Named Global Outbreak (PANGO) lineage classification using Pangolin into four categories, namely Omicron, XBB, JN.1 (SARS-CoV-2 Omicron-derived lineages designated according to the PANGO nomenclature), and other variants (Figure 1A). Sample collection time points for WTD (orange bars) and human subjects h.s.#1 and h.s.#2 (yellow and purple bars) were mapped against prevalent regional variants, confirming alignment between individual infections and regional circulation patterns, with EG.5.1.1 (XBB lineage) identified in h.s.#1 (8/31/2023) and BA.5 in h.s.#2 (08/2022) (Figure 1A).

**Figure 1:**
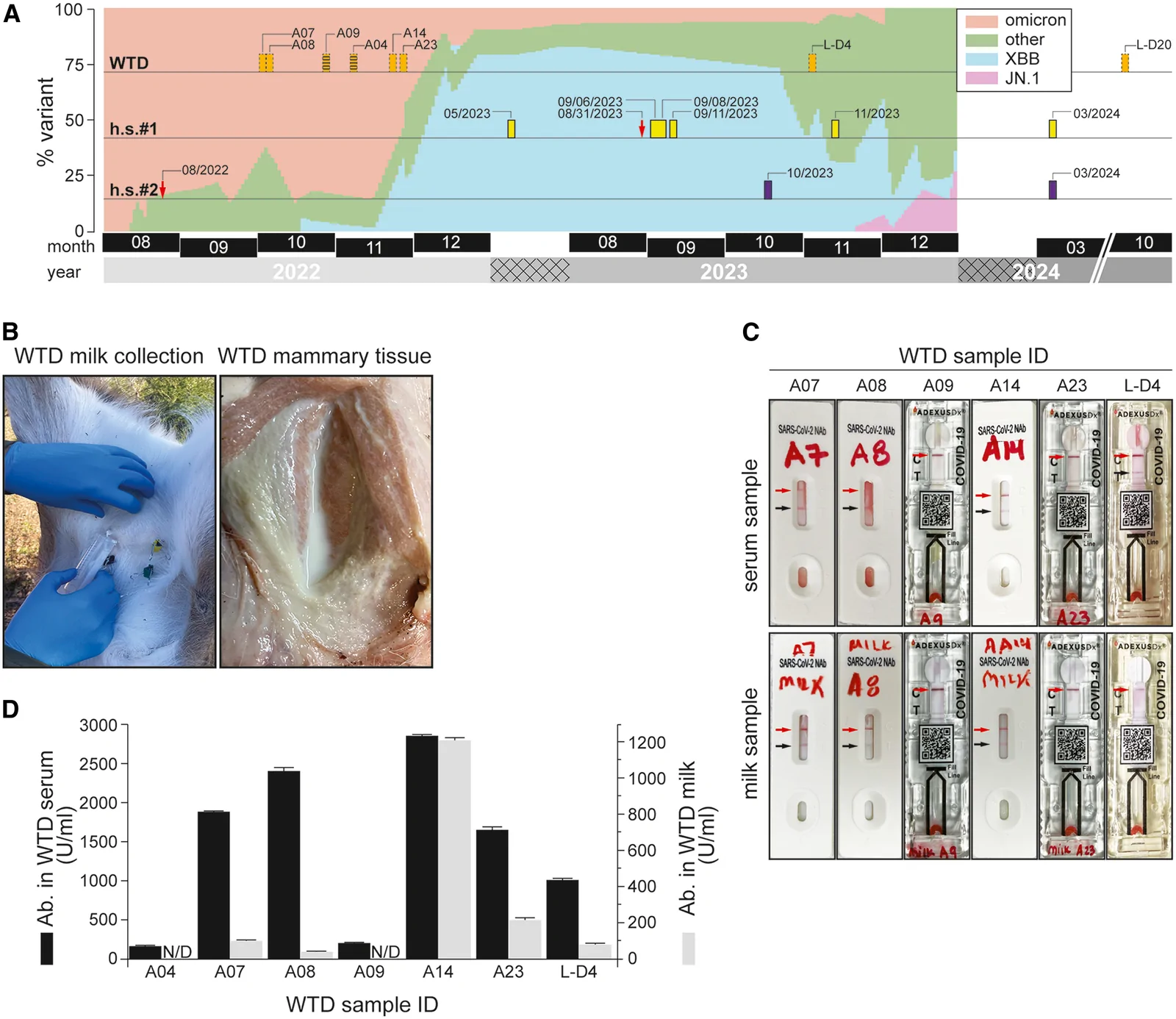
Detection of SARS-CoV-2 antibodies in white-tailed deer and human specimens (A) Deer and human specimen collection was distributed across a period spanning the Omicron, XBB, and JN.1 waves in central Virginia. Timeline showing SARS-CoV-2 variant circulation in central Virginia (2022–2024) based on whole-genome sequencing of 654 positive specimens. Variants are categorized as Omicron (orange), XBB (blue), JN.1 (pink), and other (green). Horizontal tracks indicate the source of samples WTD (top) and human subjects (middle and bottom labeled h.s.#1 and h.s.#2, respectively). Sample collection time points for deer (orange dashed bars, sample IDs [collection date: A04 (11/22), A07 (10/22), A08 (10/22), A09 (10/22), A14 (11/22), A23 (11/22), L-D4 (11/23), and L-D20 (10/24)] and human subjects (h.s.#1 in yellow, h.s.#2 in purple) are indicated relative to variant prevalence in the region. Red arrows indicate times at which the human subject sample yields a positive result by RT-qPCR. The *x* axis corresponds to the timeline (month in black and year in shades of gray) from mid-2022 through 2024 when collection took place. Crossed patterns indicate the time of the year at which collection did not occur. (B) Milk was recovered from lactating does both by manual expression in the field and from the surgically exposed mammary gland. Milk collection from a lactating doe (left) and surgically incised mammary gland displaying homogenous tissue with milk expression (right). (C) Lateral flow immunochromatography resolves SARS-CoV-2 antibody reactivity in serum and milk drawn from the same six animals. Lateral flow immunochromatography results for SARS-CoV-2 antibodies in serum (top) and milk (bottom) from six WTD specimens (A07, A08, A09, A14, A23, and L-D4). White and clear cassettes are the GenScript SARS-CoV-2 Neutralizing Antibody Rapid Test Kit and the ADEXUSDx COVID-19 Test (NOWDx, Inc.), respectively. Red arrow indicates a valid positive control test. Black arrow indicates the position of a band if positive for SARS-CoV-2 antibodies. (D) ELISA test resolves S1 RBD-specific IgG in paired WTD serum and milk. Quantitative ELISA results showing SARS-CoV-2 S1 RBD-specific IgG levels in deer serum (black bars, left axis) and milk (gray bars, right axis) from seven WTD specimens (A04, A07, A08, A09, A14, A23, and L-D4). Values represent mean ± SEM in U/mL. Samples A04 and A09 were below detection limit (N/D, <15 U/mL).

### Detection of SARS-CoV-2 antibodies in WTD and human samples

Antibody responses varied among the eight lactating does sampled. Six were screened by lateral flow immunochromatography (GenScript DXK007 and ADEXUSDx; Figure 1C) and seven were assayed by quantitative IgG ELISA (Figure 1D). Three deer (A07, A08, and A14) tested positive across all platforms in both serum and milk, with serum antibody concentrations of 691.8 ± 36.5, 2,396.8 ± 33.4, and 2,854.3 ± 7.9 U/mL, respectively, and corresponding milk concentrations of 136.8 ± 14.1, 74.7 ± 4.5, and 1,237.3 ± 147.7 U/mL. Sample L-D4 showed positive lateral flow results only in serum but demonstrated detectable IgG by ELISA in both serum (948.2 ± 13.4 U/mL) and milk (157.6 ± 22.6 U/mL). Sample A23, while negative by lateral flow assay, yielded measurable antibody levels by ELISA (serum: 630.5 ± 47.5 U/mL; milk: 227.5 ± 5.9 U/mL). Two samples (A04 and A09) remained negative across all assays. For comparative context, human milk from h.s.#1 was collected pre-infection (05/2023), during confirmed infection (08/2023), and post-infection (11/2023 and 03/2024), with post-infection IgG levels reaching 2,441.1 ± 151 U/mL (11/2023) and 488.2 ± 98 U/mL (03/2024); h.s.#2 showed undetectable milk antibodies one year after infection, consistent with time-dependent waning reported for human milk antibodies^19^^,^^20^ (Figure S2A). These results provide the first evidence of SARS-CoV-2-specific neutralizing antibodies in the milk of a wildlife species, demonstrating that lactating WTD mount humoral immune responses detectable in both serum and milk.

### Viral RNA detection

SARS-CoV-2 RNA was detected in nasopharyngeal swabs from h.s.#1 during active infection (08/31/2023, 09/06/2023, and 09/08/2023) and from h.s.#2 (08/2022) but was absent in milk samples collected at corresponding time points (Figure S2B). Swabs collected before or after infection yielded negative RT-qPCR results. Viral particles were not detected in nasal swabs or milk from any WTD by either RT-qPCR or transcription-mediated amplification (TMA, Hologic Panther^21^), indicating no evidence of active viral shedding at the time of sampling despite the presence of neutralizing antibodies.

### Histopathological analysis

Mammary gland tissue examination in WTD revealed differences between physiological states (Figure S2C). Immunohistochemistry detected rare SARS-CoV-2-positive histiocytes in WTD mammary tissue, consistent with incidental histiocytic migration rather than primary mammary gland infection, with no evidence of active viral replication in mammary epithelial cells. The presence of SARS-CoV-2-positive histiocytes, however rare, represents a potential source of cell-associated viral particles in milk, motivating our investigation of intrinsic antiviral properties of WTD milk under controlled conditions.

### Temperature-dependent viral stability analysis

Three SARS-CoV-2 variants representing distinct evolutionary stages were selected for *in vitro* studies, representing PANGO lineage A (original), B.1.1.7 (Alpha), and BA.1.1.529 (Omicron).^2^^,^^6^ In human milk, all three inactivated variants remained stable at room temperature and 37°C, with Cq values maintained below the experimental cutoff throughout the 60 min incubation period (Figures 2Ai, 2Aii, 2Bi, and 2Bii). Conversely, WTD milk exhibited rapid degradation of all variants under identical conditions, with viral copy numbers declining precipitously within the first 10 min and falling below detectable levels (Cq > 40) within 30–40 min at physiological temperatures (Figures 2Bi and 2Bii). At 63°C, both milk types showed rapid viral RNA degradation within 20–30 min, though WTD milk exhibited faster initial degradation kinetics (Figures 2Aiii and 2Biii). Rapid RNA degradation in WTD milk was consistent across individual animals and collection time points, WTD samples A23 and L-D4, collected approximately one year apart, demonstrated similar degradation patterns for all three inactivated variants, as did human milk from h.s.#1 collected pre- and post-infection (Figure 3A), indicating that differential viral stability reflects intrinsic species-specific milk properties rather than individual variation.

**Figure 2:**
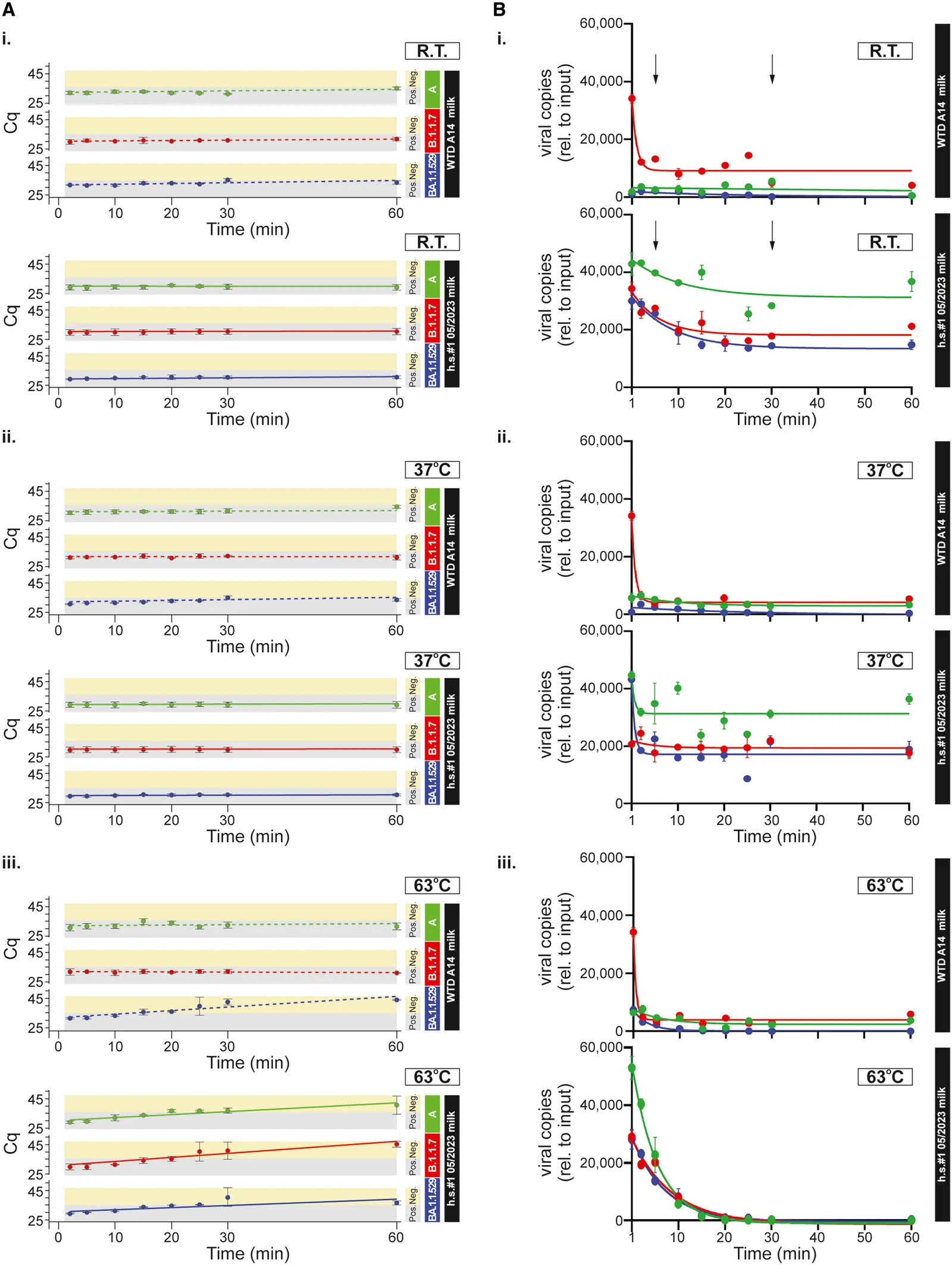
Thermal stability of SARS-CoV-2 variants in human and WTD milk (A) Cq time courses track the persistence of detectable SARS-CoV-2 RNA variants in human and WTD milk across three incubation temperatures. Time-course RT-qPCR analysis of SARS-CoV-2 variant stability in milk at room temperature (RT, 22°C, i), 37°C, (physiological temperature, ii) and 63°C (pasteurization temperature, iii). Human and WTD milk samples were spiked with A (green), B.1.1.7/Alpha (red), or BA.1.1.529/Omicron (blue) variants and incubated for up to 60 min. Cq values indicate remaining viral RNA levels over time, with higher Cq values representing lower viral loads. For each experiment, the cutoff threshold value for the detection of the N gene separates regions for which Cq values indicate a positive versus a negative result (gray- and yellow-shaded areas, respectively). Each plotted point is represented as the mean Cq ± SD. A linear relationship has been drawn between the points within each image to show progressive degradation. Cq cutoff for the N gene was 36.73 ± 0.74. (B) Absolute copy number measurements quantify the magnitude and kinetics of viral RNA loss in WTD and human milk at the same three temperatures. Quantitative viral copy analysis showing viral RNA stability expressed as copies relative to input over time. (i–iii) Incubation temperatures of RT (22°C, 37°C, and 63°C), respectively. Upper images show WTD A14 milk; lower images show human h.s.#1 (05/2023) milk. Viral variants are color-coded as in (A): A (green), B.1.1.7/Alpha (red), and BA.1.1.529/Omicron (blue). Data points represent mean viral copies ±SD.

**Figure 3:**
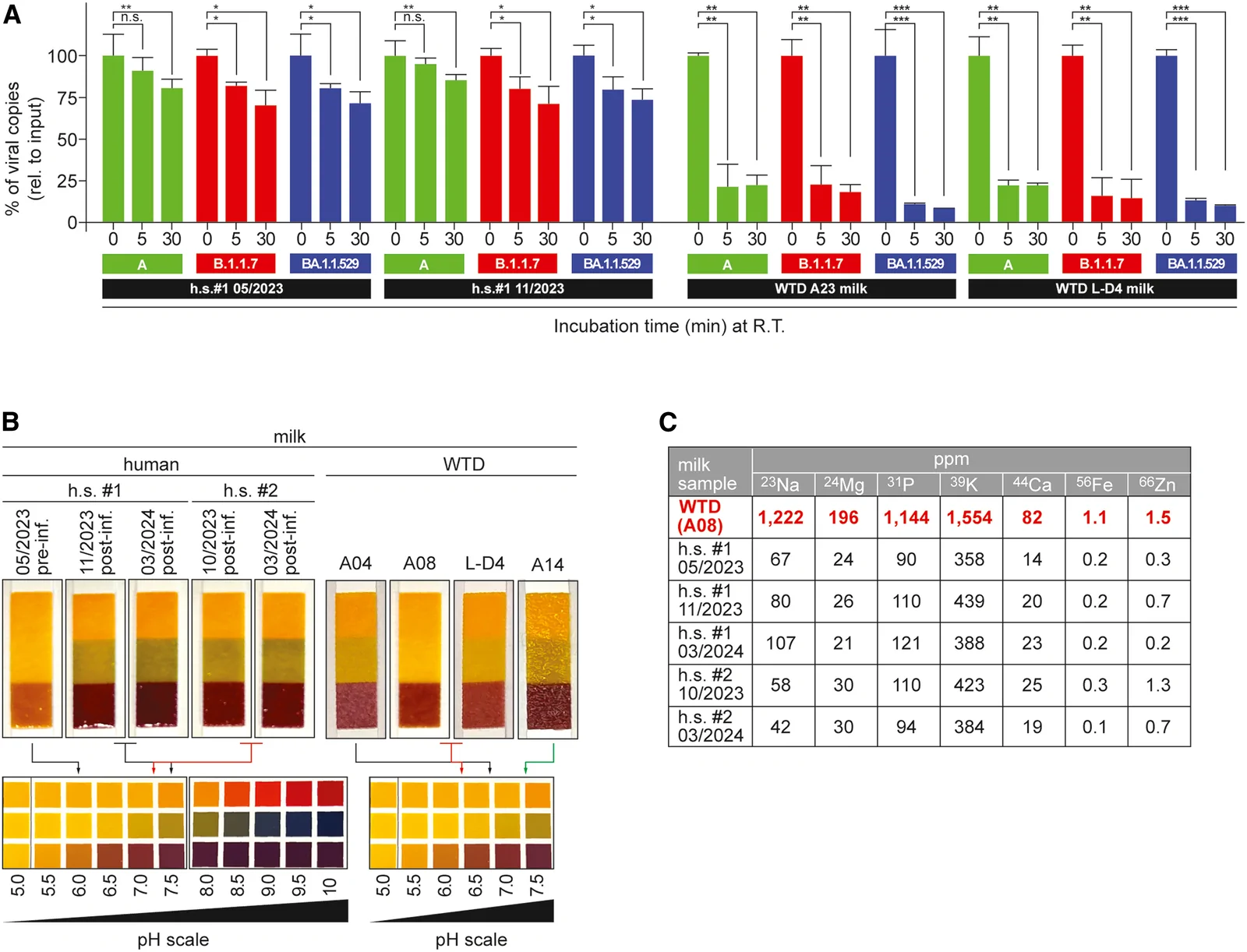
Comparative viral RNA stability and biochemical characterization of WTD and human milk (A) Viral RNA recovery after room temperature incubation was substantially lower in WTD milk than in human milk for all three variants. Comparative viral RNA stability in human and WTD milk. Samples were spiked with A (green), B.1.1.7/Alpha (red), or BA.1.1.529/Omicron (blue) variants and incubated at room temperature for 5- or 30-min. Results show percent viral RNA recovery relative to input. Human milk (h.s.#1 05/2023, h.s.#1 11/2023) maintained higher RNA stability compared to WTD samples (A23, L-D4). Bar graphs show mean ± SD of three technical replicates. One-way ANOVA with Holm-Sidak post hoc statistical significance: ∗∗*p* < 0.01, ∗∗∗*p* < 0.001, n.s., not significant. (B) Strip colorimetry resolves the pH of individual human and WTD milk samples, including longitudinal samples from both human subjects. pH determination of human and WTD milk samples using colorimetric indicator strips. Color changes correspond to pH values ranging from 5.0 to 10 as indicated by the color scale. Human milk samples (h.s.#1, h.s.#2) and WTD samples (A04, A08, L-D4, A14) are shown. Dates indicate the time at which the corresponding milk sample was collected based on the information displayed in Figure 1A. For each milk sample, the pH test strip (upper strip) is shown alongside the manufacturer’s reference color scale (lower strip) to allow visual comparison. The strip color for each sample was matched to the reference scale, and the pH was recorded to the nearest 0.5 unit based on the best color match. (C) ICP-MS shows that WTD milk contains higher concentrations of all seven measured minerals than any human milk sample examined. Elemental composition analysis by ICP-MS showing concentrations (ppm) of seven key minerals (Na, Mg, P, K, Ca, Fe, and Zn) in human milk samples (h.s.#1 at multiple time points, h.s.#2) and WTD milk (A08). Values in red indicate ≥5-fold higher concentrations in WTD milk compared to human milk.

### Biochemical characterization of WTD and human milk

Human milk from h.s.#1 ranged from pH 6.0 pre-infection to approximately 7.25 post-infection, while WTD milk displayed neutral to slightly alkaline values across all samples (Figure 3B). Ketone body analysis confirmed normal metabolic profiles in both species, ruling out ketosis-related contributions to the observed stability differences (Figure S3A). Inductively coupled plasma mass spectrometry (ICP-MS) mineral analysis revealed markedly elevated concentrations across seven key elements in WTD milk (sample A08) relative to human milk, including sodium (1,222 ppm), magnesium (196 ppm), phosphorus (1,144 ppm), and potassium (1,554 ppm) representing 5- to 20-fold elevations, with iron, calcium, and zinc similarly elevated (Figure 3C). Human milk mineral profiles remained remarkably consistent across all time points and subjects regardless of infection status, highlighting these differences as intrinsic species characteristics. The striking elevation in ionic concentrations in WTD milk prompted us to investigate their contribution to viral instability through direct mineral supplementation experiments. Total protease activity was 3- to 5-fold higher in WTD milk samples (A08, A09, A14, and L-D4) compared to human milk at all pH values tested, with peak activity at pH 8 in both species (Figure S3B). Notably, sample A23 exhibited protease activity levels comparable to human milk yet achieved complete viral RNA degradation (Figure 3A), suggesting that proteolytic activity alone does not account for the antiviral phenotype and that ionic concentrations may provide compensatory antiviral capacity, motivating the combinatorial mechanistic experiments described in the subsequent sections.

### Mineral supplementation effects on SARS-CoV-2 stability

To test whether elevated ionic concentrations contribute to viral instability, human milk (h.s.#1, 05/2023) was supplemented with individual minerals at concentrations equivalent to those found in WTD milk and spiked with each inactivated variant across three temperature conditions (Figure 4A). Individual mineral supplementation revealed pronounced variant-specific susceptibilities, wherein B.1.1.7 demonstrated significant sensitivity to all tested minerals across all temperatures, while variants A and BA.1.1.529 maintained comparatively higher viral copy numbers under identical ionic conditions (Figure 4A). These variant-specific responses suggested that mutation-driven differences in viral surface electrostatics underlie differential ionic susceptibility and motivated the combinatorial protease inhibition experiments to dissect the relative contributions of ionic and proteolytic mechanisms.

**Figure 4:**
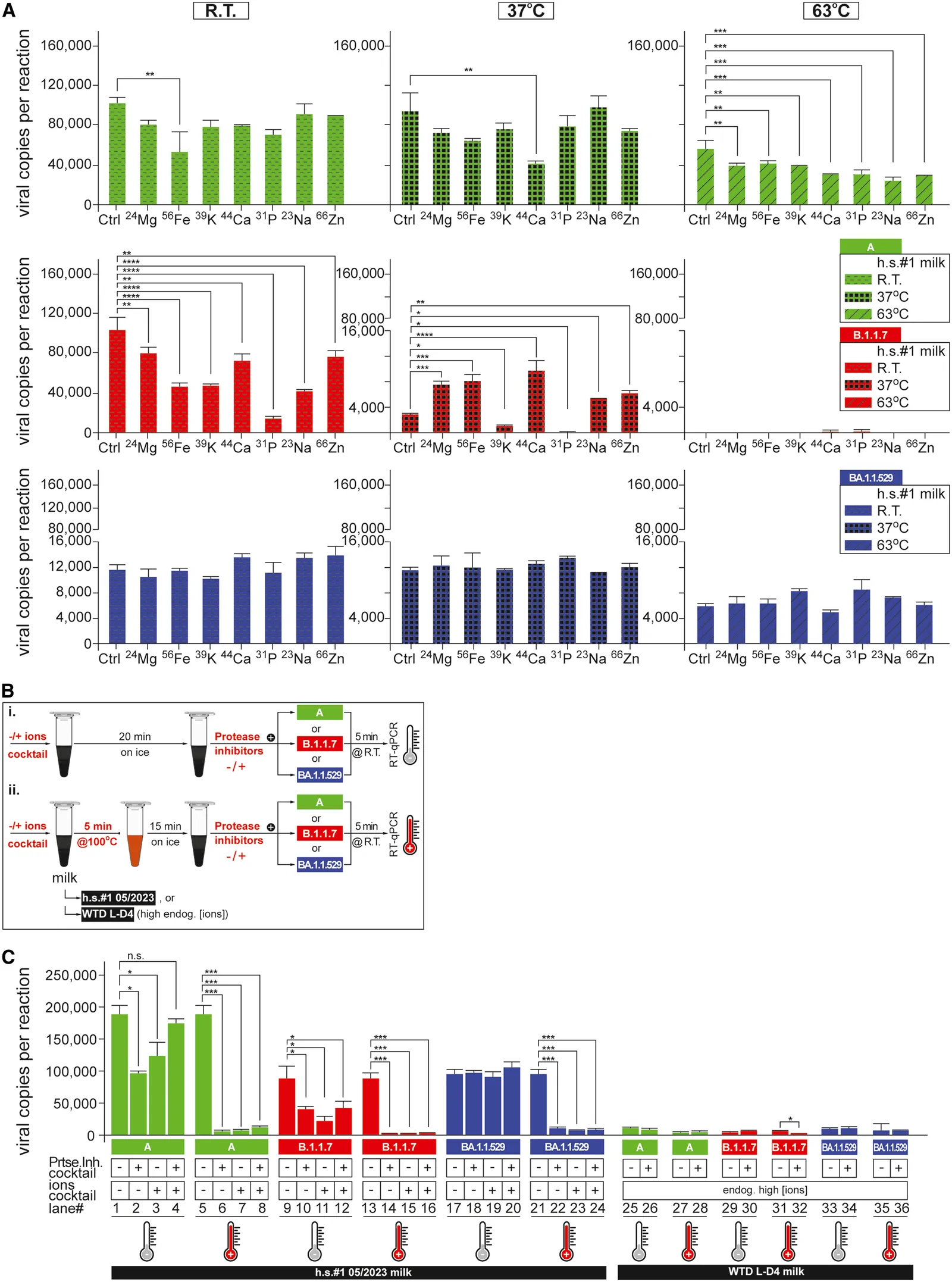
Mineral supplementation influences SARS-CoV-2 variant stability in milk (A) Supplementation of human milk with individual ions altered viral copy recovery in a manner dependent on both the variant and the incubation temperature. Human milk (h.s.#1 05/2023) was supplemented with individual ions (^23^Na, ^24^Mg, ^31^P, ^39^K, ^44^Ca, ^56^Fe, and ^66^Zn) at concentrations equivalent to those found in WTD milk as described in the STAR Methods. Samples were spiked with A (green), B.1.1.7/Alpha (red), or BA.1.1.529/Omicron (blue) variants and incubated at room temperature (RT, 22°C), 37°C, or 63°C. Viral copy recovery is shown as copies per reaction (mean ± SD). Control (Ctrl) represents unsupplemented human milk. (B) Schematic of the two workflows used to separate the contributions of ion supplementation and protease inhibition, with and without a heat step. Step-by-step experimental workflow diagram for assessing viral RNA degradation in milk under various conditions (e.g., ion supplementation, protease inhibitors). (i) Human milk (h.s.#1) was supplemented, or not, with ion cocktails to mimic the levels found in WTD milk, maintained for 20 min on ice, and then treated, or not, with protease inhibitors before spiking with the indicated virus and performing RT-qPCR analysis as described in the STAR Methods. For WTD milk (L-D4), endogenous ion levels exist and only the effect of protease inhibitor addition was monitored. In (ii), the experimental protocol includes an intermediate step where samples are incubated at 100°C for 5 min followed by 15 min on ice before adding, or not, protease inhibitors and proceeding as in (i). (C) Protease inhibition and ion supplementation produced condition-dependent changes in viral copy recovery in human milk. Values are mean ± SD of three independent measurements. Effects of protease inhibition and ion supplementation on viral RNA degradation. Human milk (h.s.#1 05/2023) and WTD milk (L-D4) were treated with combinations of protease inhibitor cocktail and ion mixture, then spiked with viral variants as indicated in (B). Bar graphs show viral copies per reaction under different conditions (mean ± SD). Temperature symbols indicate room temperature (gray thermometer) or heat treatment at 100°C (red thermometer). In all cases, one-way ANOVA was performed with Holm-Sidak post hoc, statistical significance is indicated as: ∗*p* < 0.05, ∗∗*p* < 0.01, ∗∗∗*p* < 0.001, ∗∗∗∗*p* < 0.0001, n.s., not significant.

The reduced viral copy numbers observed under elevated ionic conditions reflect genuine RNA degradation rather than RT-qPCR inhibition or extraction inefficiency, as supported by three independent lines of evidence. The variant-specific patterns of RNA degradation, B.1.1.7 exhibiting pronounced sensitivity while variants A and BA.1.1.529 maintained comparatively higher copy numbers under identical ionic conditions, are incompatible with non-specific matrix-mediated inhibition, which would affect all targets uniformly. Protease inhibitor addition partially rescued viral copy numbers in ion-supplemented milk (Figure 4C), a result that cannot be explained by irreversible assay inhibition. Furthermore, endogenous WTD milk, despite its substantially higher ionic concentrations, yielded consistent and reproducible Cq values for spiked virus at time zero across independent experiments, confirming that the RT-qPCR assay tolerates the ionic range tested.

### Mechanistic dissection of ionic and proteolytic contributions to viral degradation

Controlled *in vitro* experiments combining ion supplementation and protease inhibition (Figure 4B), revealed that protease inhibitors alone did not prevent degradation of A and B.1.1.7 variants in human milk (Figure 4C, lanes 1 vs. 2 and 9 vs. 10) but stabilized BA.1.1.529 (Figure 4C, lanes 17 vs. 18). Ion cocktail addition further reduced B.1.1.7 stability (Figure 4C, lanes 1 vs. 3), an effect partially reversed by subsequent protease inhibitor addition (Figure 4C, lanes 1 vs. 4), demonstrating synergistic contributions of ionic stress and proteolytic activity to viral degradation. In WTD milk (L-D4), protease inhibitors provided only modest protection while substantial degradation persisted (Figure 4C, lanes 25–26, 29–30, 33–34), confirming that endogenous ionic concentrations alone promote viral instability independently of proteolytic activity. Unexpectedly, heat pretreatment (100°C, 5 min) substantially enhanced viral RNA degradation across all inactivated variants and both milk types (Figure 4C, lanes 5–8, 13–16, 21–24, 27–28, 31–32, and 35–36), indicating that heat-induced structural changes in the milk matrix might activate additional degradative mechanisms beyond enzymatic activity.

### Enzymatic profiling and antimicrobial factor analysis

API-ZYM (a semiquantitative micromethod strip for the simultaneous screening of enzymatic activities; bioMérieux) profiling, which measures total enzymatic capacity in unfractionated biological fluids irrespective of cellular origin, revealed distinct enzymatic landscapes in human and WTD milk (Figure 5A). Human milk exhibited broad enzymatic activity including esterases, phosphatases, lipases, and glycosidases, with partial retention after heat treatment. WTD milk showed strong activity for alkaline phosphatase, esterases (C4, C8), lipase (C14), leucine arylamidase, and glycosidases, but complete loss of all enzymatic activity following heat treatment (Figure 5A), contrasting with human milk and implicating non-enzymatic factors in the continued viral degradation observed in heated WTD milk samples. WTD milk demonstrated substantially higher baseline lactoperoxidase (LPO) activity (∼40 U/μL·min) versus human milk (<0.01 U/μL·min), with residual activity persisting after heat treatment (Figure 5B). Lysozyme activity was higher in WTD milk (345 mU/mL·min) than human milk (193 mU/mL·min) at room temperature but converged after heat treatment (Figure 5C), ruling out lysozyme as the primary driver of differential antiviral activity. Lactoferrin levels were comparably high in both species (∼590 μg/mL human; ∼490 μg/mL WTD) and dramatically reduced by heat treatment in both (Figure 5D). Supplementation of human milk with suboptimal LPO (0.4 U/μL, ∼10-fold below endogenous WTD levels) combined with the ion cocktail produced synergistic antiviral activity across all three variants, mimicking the RNA degradation pattern of native WTD milk and confirming that ionic concentrations potentiate LPO-mediated viral RNA degradation (Figure 5E).

**Figure 5:**
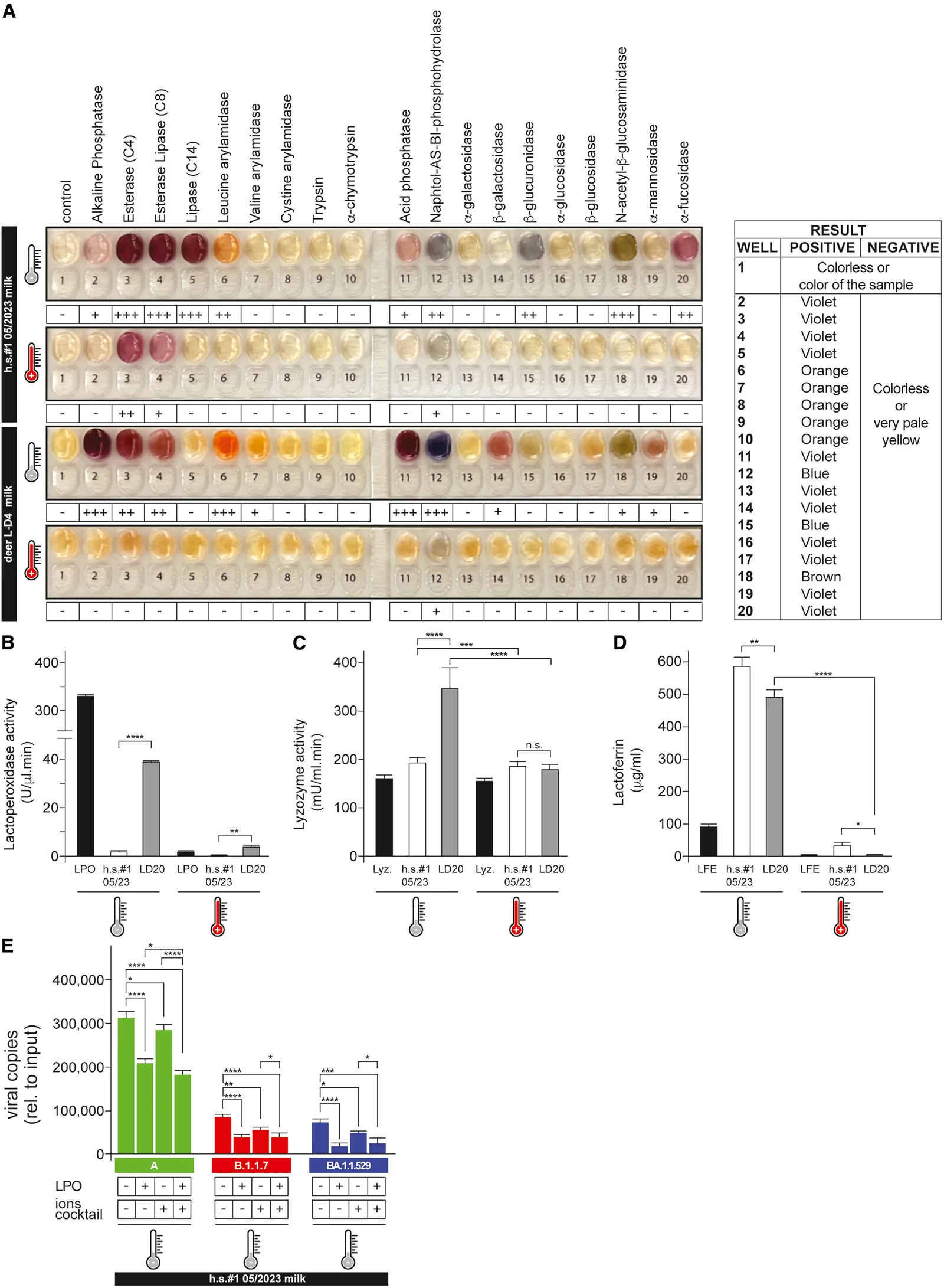
Enzymatic activity profiling and antiviral component characterization in milk (A) API-ZYM profiling resolves distinct enzymatic activity patterns in human and WTD milk and the loss of most activities after heating. API-ZYM enzymatic activity strips showing 20 different enzyme activities in human milk (h.s.#1 05/2023) and WTD milk (L-D4) at room temperature (gray thermometer) and after heating to 100°C (red thermometer). Color intensity indicates enzyme activity levels: negative (−), weak (+), moderate (++), or strong (+++). Enzymes detected include alkaline phosphatase, esterases, lipases, aminopeptidases, glycosidases, and others as labeled. (B) Lactoperoxidase (LPO) activity was higher in WTD milk than in human milk and was reduced by heating in both. Lactoperoxidase activity was measured in human milk (h.s.#1 05/2023), WTD milk (L-D20), and commercial LPO standard before and after heat treatment (100°C). Activity is expressed as U/μL·min with error bars showing mean ± SD. One-way ANOVA within each temperature with Holm-Sidak post hoc, statistical significance: ∗∗*p* < 0.01, ∗∗∗∗*p* < 0.0001. (C) Lysozyme activity was higher in WTD milk than in human milk before heating, and the difference was not apparent after heating. Lysozyme activity in the same samples and a commercial lysozyme control was measured using fluorometric assay. Activity is expressed as mU/mL·min before and after heat treatment. Multiple *t* test significance: ∗∗∗*p* < 0.001, ∗∗∗∗*p* < 0.0001, n.s., not significant. (D) Lactoferrin was abundant in both human and WTD milk before heating and fell substantially after heat treatment. Lactoferrin concentration was measured by ELISA in human and WTD milk samples and commercial lactoferrin standard before and after heat treatment. Concentrations are expressed as μg/mL with error bars showing mean ± SD. Multiple *t* test significance: ∗*p* < 0.05, ∗∗*p* < 0.01, ∗∗∗∗*p* < 0.0001, n.s., not significant. (E) Addition of lactoperoxidase, with or without the ion cocktail, lowered viral copy recovery in human milk for all three variants. Functional validation of LPO-mediated viral RNA degradation. Human milk (h.s.#1 05/2023) was supplemented with LPO and/or ion cocktail, then spiked with A (green), B.1.1.7/Alpha (red), or BA.1.1.529/Omicron (blue) variants. Bar graphs show mean ± SD of viral RNA copies recovery relative to input. One-way ANOVA was performed with Holm-Sidak post hoc, statistical significance: ∗*p* < 0.05, ∗∗*p* < 0.01, ∗∗∗*p* < 0.001, ∗∗∗∗*p* < 0.0001, n.s., not significant.

## Discussion

This study provides the first characterization of SARS-CoV-2 dynamics in wildlife milk, revealing that WTD mammary secretions harbor neutralizing antibodies and possess intrinsic multifactorial antiviral properties that promote rapid degradation of viral RNA through synergistic biochemical mechanisms distinct from those operating in human milk. The convergence of humoral immunity, restriction of viral infection to inflammatory histiocytes rather than mammary epithelial cells, and rapid instability of free viral particles within the WTD milk matrix collectively indicate that lactation in this major wildlife host constitutes a pathway of pathogen clearance rather than maternal-offspring transmission, a finding that reframes the biological role of milk in the context of viral persistence within this species.

The absence of detectable viral RNA in WTD milk despite serological evidence of prior infection is consistent with observations in human milk, where viral RNA is rarely detected even during active infection.^13^^,^^22^ This pattern of antibodies being present and infectious virus absent, is not self-evident. It reflects an active immunological and biochemical environment rather than simple absence of mammary tropism. It contrasts markedly with pathogens for which milk-borne transmission is well-established, as HIV-1 exploits activated mammary CD4^+^ T cells as a stable viral reservoir^23^^,^^24^ and, in settings without effective prophylaxis, breastfeeding accounts for more than half of pediatric HIV infections globally.^25^ Similarly, Rift Valley fever virus demonstrates prolonged infectivity in ruminant milk with documented transmission through raw milk consumption.^17^^,^^18^ The contrast underscores that milk-based transmission risk is simultaneously pathogen-specific and species-specific, and cannot be inferred from general principles, reinforcing the need for independent empirical characterization of each pathogen-host-matrix combination.

Sensitivity to ionic stress differs among lineages. B.1.1.7 was the lineage most destabilized by individual mineral supplementation, whereas lineage A and BA.1.1.529 retained higher copy numbers under the same ionic conditions (Figure 4A). In native WTD milk, where the ionic, proteolytic, and enzymatic arms operate together, this variant distinction is not preserved, and degradation of B.1.1.7 and BA.1.1.529 appears more rapid than that of lineage A (Figures 2B and 3A). Ionic sensitivity therefore identifies the route by which a lineage is degraded rather than the magnitude of its net susceptibility to the matrix. Computational analyses demonstrate that Omicron variants carry markedly more positive electrostatic potential in the receptor-binding domain than ancestral strains, driven by multiple charge-increasing mutations,^26^ a finding corroborated by adaptive Poisson-Boltzmann calculations showing that positive surface potential on the spike protein increased progressively from the original A strain through successive variants,^27^ while B.1.1.7 displays a comparatively smaller electrostatic shift, a structural profile that may confer particular vulnerability to divalent cation interference. These findings are consistent with studies demonstrating that ionic strength modulates electrostatic interactions and virus-surface behavior across diverse viral systems.^28^^,^^29^

These distinctions have a bounded epidemiological reach. B.1.1.7 is the lineage most sensitive to ionic stress in our assays and the lineage recovered from free-ranging WTD more than a year after it ceased to circulate in humans.^30^^,^^31^^,^^32^ The two observations are compatible, since the milk matrix acts only at the maternal-offspring interface and only during the bounded interval of lactation, whereas persistence in WTD is sustained by respiratory transmission among all age classes. Reported seasonality reinforces this separation, since prevalence in WTD is highest during winter and spring,^32^ when lactation has ceased. Lineage-specific susceptibility to milk biochemistry is thus expected to shape viral fate within a defined seasonal and anatomical compartment rather than to determine which lineages persist in the population. As SARS-CoV-2 continues to evolve, variant-driven shifts in susceptibility to host milk biochemistry represent a variable that strain-agnostic assessments of viral persistence in biological matrices are unlikely to capture.

The multifactorial nature of antiviral activity in WTD milk, evidenced by complete viral RNA elimination in specimen A23 despite protease levels comparable to human milk, demonstrates that no single mechanism fully accounts for the antiviral phenotype. Rather, synergistic interactions among ionic stress, proteolytic activity, and LPO, whose activity is potentiated by the elevated ionic environment, create a biochemical threshold wherein multiple pathways provide compensatory antiviral capacity. This is consistent with the demonstrated ability of the LPO/H_2_O_2_/SCN^−^ system to inactivate influenza A and B virus strains with strain-dependent substrate specificity,^33^ establishing LPO-mediated oxidation as a broad-spectrum antiviral mechanism across respiratory virus families. Critically, functional reconstitution of human milk with LPO at concentrations approximately 10-fold below endogenous WTD levels, combined with an ionic cocktail mimicking WTD mineral composition, recapitulated the antiviral phenotype across all three variants (Figure 5E). This gain-of-function result demonstrates that the ionic-enzymatic axis is sufficient to convert a permissive matrix into a restrictive one, and that ionic potentiation of LPO activity underlies the species-specific difference in viral fate. The paradoxical enhancement of viral degradation following heat pretreatment, despite elimination of detectable enzymatic activity by API-ZYM in WTD milk, implicates non-enzymatic degradative mechanisms. Plausible explanations include heat-induced reorganization of the protein matrix exposing viral RNA to sequestered degradative factors, thermal release of chelated divalent cations increasing effective free ion concentrations, generation of reactive Maillard intermediates, and activation of thermally modulated ribonucleases.^34^^,^^35^ Notably, heat treatment of ruminant milk produces species-specific effects on protease activity, with 85°C treatment increasing activity by 68% in the bovine casein fraction while decreasing it in bovine milk,^34^ indicating that thermal modulation of degradative capacity is itself species-dependent. This study did not include direct RNase assays or RNase inhibition controls and the protease inhibitor cocktail employed targets serine, cysteine, and aspartic proteases but not RNase activity, and therefore the relative contribution of endogenous ribonucleases to RNA degradation remains to be formally quantified. Resolving these mechanisms will require RNase inhibition controls, quantification of free versus protein-bound ion fractions, and orthogonal RNA integrity assessments. The non-linear thermal behavior observed here carries practical relevance, since conventional bovine milk pasteurization parameters cannot be assumed to predict viral behavior in cervid milk, whose ionic and enzymatic landscape differs fundamentally.^15^^,^^36^

The demonstrated transferability of antiviral capacity from WTD to human milk through defined biochemical supplementation carries practical implications in two domains. For the emerging cervid dairy industry, where elk and deer milk are increasingly marketed for human consumption,^36^ the distinct mineral and enzymatic profiles documented here, together with the non-linear thermal behavior described previously, underscore the need for species-specific pathogen safety protocols rather than extrapolation from bovine parameters. For translational research, the identification of LPO as a rate-limiting enzymatic factor whose activity is potentiated by physiologically relevant ionic concentrations provides a defined, naturally occurring antiviral axis amenable to formulation studies.

SARS-CoV-2 dynamics in WTD followed a distinctive temporal course. At landscape scale, local epidemics in WTD were well approximated by a single outbreak peak followed by fade-out, with local effective reproduction ratios between 1 and 2.5, and 10% of variability in peak prevalence explained by human infection pressure.^37^ Following the marked rise documented between the 2020 and 2021 hunting seasons,^30^ surveillance recorded declines in viral detection between the 2021 and 2022 seasons,^38^ coincident with the displacement of earlier lineages by Omicron in humans. Our observations bear on this pattern through two mechanisms whose weight increased over the course of the epidemic.

The first is immunological. The detection of neutralizing antibodies in WTD milk identifies a maternal immune conduit whose significance for SARS-CoV-2 dynamics in wildlife populations has not been examined. Antibodies only enter milk after maternal exposure, so milk from a naive doe delivers no antibodies, whereas milk from an exposed doe delivers antibodies at the concentrations reported here, reaching 1,237 U/mL in individual animals (Figure 1D). In ungulates, systemic passive immunity depends on the absorption of intact colostral immunoglobulins across the proximal small intestine during the first one to two days of life, after which gut closure ends macromolecular transfer.^39^ In cervids, immunoglobulin concentration falls steeply across this transition, and milk collected more than two weeks after parturition is compositionally mature.^40^ The specimens analyzed here were mature milk rather than colostrum, establishing that exposed does secrete SARS-CoV-2 antibodies into the mammary compartment throughout lactation without establishing the magnitude of systemic transfer to fawns. Experimental SARS-CoV-2 infection of WTD is subclinical,^1^ and clinical disease is rarely reported in free-ranging animals, so the relevant consequence of maternal antibody in this host is not protection from disease but a reduction in the susceptibility and shedding of individual fawns.^41^ Whether antibodies transferred via WTD milk reduce infection or shedding in nursing fawns remains to be directly demonstrated.

WTD are synchronous seasonal breeders, and fawning introduces a discrete annual cohort of immunologically naive individuals within a narrow window.^42^ Maternally derived antibodies modulate viral dynamics in other seasonally breeding wildlife systems.^43^^,^^44^ The immunity of each successive cohort upon entering the population would therefore reflect the exposure history of the does that nursed it. Cohorts born during 2020 and early 2021 would have entered the autumn transmission season without maternally derived immunity, whereas cohorts born after the extensive infection documented across WTD populations during 2021^30^ would have encountered a maternal antibody pool. This pool is a function of cumulative exposure within the deer population rather than of the identity of the circulating lineage, and it accrued over the same interval as the transition to Omicron in humans. The temporal coincidence of the two does not require Omicron-specific restriction at the maternal-offspring interface.

The second is biochemical. As described previously, susceptibility to the WTD milk matrix increased for lineages that diverged from the ancestral strain, with B.1.1.7 and BA.1.1.529 degraded more rapidly than lineage A (Figures 2B and 3A). The lineages that seeded the earliest deer epidemics are therefore the most stable at the maternal-offspring interface.

Taken together, these observations support a working model in which milk constrains amplification within the annual pulse of naive animals, through a maternal antibody pool that accrued with population exposure and a matrix that restricts post-ancestral lineages more efficiently than lineage A. Several considerations limit this model. Our panel does not include Delta, which contributed substantially to infections in WTD during 2021, and B.1.1.7 and BA.1.1.529 are not separable in our assays, so the biochemical data support increased matrix susceptibility relative to lineage A rather than an Omicron-specific effect. We did not measure antibody in fawns, demonstrate intestinal absorption of milk-derived immunoglobulin, or assess functional protection. A temporal constraint applies as well. Hunter-harvest surveillance occurs on average approximately 148 days after median parturition,^45^ by which time milk is compositionally mature.^40^ Any contribution of milk would therefore be registered indirectly, through the condition of the fawn cohort entering the autumn. Discriminating this model from population-level explanations such as depletion of susceptible animals^37^ and species- and variant-specific differences in ACE2-spike compatibility among cervids^46^ requires paired sampling of does and fawns across lactational stages, a sampling design not represented in the present dataset and beyond the scope of this study.

### Limitations of the study

This study is subject to limitations that define the boundaries of interpretation. The use of chemically inactivated virus, while standard in milk stability studies under BSL-2 conditions,^13^ does not recapitulate the behavior of replication-competent virus, and the RT-qPCR readout measures viral RNA degradation rather than loss of infectivity. Consequently, “inactivation” as used here refers to destruction of viral RNA integrity rather than demonstrated loss of replicative capacity; and live virus validation with infectivity-based readouts such as plaque or TCID50 assays would be required to establish correspondence between RNA degradation and infectious potential. The sample size (eight lactating does from three counties across three hunting seasons) is sufficient for mechanistic characterization but insufficient for population-level generalization across WTD’s broad geographic range. Similarly, the human cohort (*n* = 2 lactating subjects) constrains cross-species comparisons, although the consistency of mineral and protease profiles across all human time points supports their representativeness. Sampling was constrained to the hunting season, which captures late-lactation animals, and milk composition in cervids is not static across lactation.^15^ Mammary tight junction integrity is compromised as lactation advances toward involution,^47^ and the resulting paracellular leakage raises milk sodium and the sodium to potassium ratio, while lowering potassium levels.^48^ Our mineral data do not follow that pattern. Sodium and potassium are elevated to a comparable degree in WTD milk relative to human milk (Figure 3C), whereas selective barrier leakage would raise sodium and depress potassium. The ionic environment described here is therefore unlikely to arise from late-lactation barrier compromise, although we cannot exclude that it is accentuated by it. Whether LPO activity, protease activity, and antibody titer are maintained, attenuated, or enhanced at earlier lactational stages remains unresolved. While functional reconstitution experiments support the mechanistic sufficiency of the ionic-proteolytic-LPO axis, they do not exclude contributions from additional bioactive components that merit independent investigation. Future studies should expand sampling across populations, lactational stages, and regions; investigate SARS-CoV-2 dynamics in other cervid species; and test whether milk-derived antibodies protect against altered infectivity in suckling fawns; and characterize thermal behavior under commercially relevant processing protocols, consolidating the mechanistic framework for understanding viral fate in species-specific biological matrices.

## Resource availability

### Lead contact

Requests for further information on resources and reagents should be directed to and will be fulfilled by the lead contact, Carla V. Finkielstein (finkielc@vt.edu).

### Materials availability

This study did not generate new unique reagents. Remaining specimens are available from the lead contact upon reasonable request and subject to material availability.

### Data and code availability

SARS-CoV-2 genome sequences generated during regional variant surveillance have been deposited at the Global Initiative on Sharing All Influenza Data (GISAID) database and NCBI and are publicly available as of the date of publication. Accession numbers are listed in Table S1. Additional data reported in this article will be shared by the lead contact upon request. This article does not report original code.

## Acknowledgments

We thank members of the Molecular Diagnostics Laboratory and Fralin Biomedical Research Institute for feedback, the hunters and private landowners for WTD harvest, and the staff at Pathology Consultants of Central Virginia. Funding was provided by USDA-10.13039/100009168APHIS and the 10.13039/100031013Fralin Biomedical Research Institute (to C.V.F.), Pathology Consultants of Central Virginia (to D.S.R), and a 10.13039/100008457University of Virginia 10.13039/100020950Department of Pathology research stipend (to A.P.B.). Funders had no role in study design, data collection, analysis, or manuscript writing.

## Author contributions

Conceptualization, D.S.R. and C.V.F.; methodology, W.S., A.C., K.L.B., M.G.U., C.R., D.M., and D.G.S.C.; formal analysis, I.A., A.C., K.L.B., M.G.U., C.R., D.M., and D.G.S.C.; investigation, L.P.T., W.S., H.A.R., A.P.B., and D.S.R.; resources, H.A.R. and D.S.R.; writing – original draft, L.P.T., C.V.F and D.S.R.; writing – review and editing, C.V.F.; supervision, C.V.F.; funding acquisition, C.V.F.

## Declaration of interests

The authors declare no competing interests.

## Declaration of generative AI and AI-assisted technologies in the manuscript preparation process

AI-assisted tools were used only for language editing and improving the clarity of the manuscript. All scientific content, interpretations, and conclusions were developed by the authors and carefully reviewed and verified.

## STAR★Methods

### Key resources table

**Table:** 

REAGENT or RESOURCE	SOURCE	IDENTIFIER
**Antibodies**		

Mouse anti-SARS-CoV/SARS-CoV-2 nucleocapsid (Clone 05)	Sino Biological	Cat# 40143-MM05; RRID: AB_2827977
Recombinant Protein A/G	Thermo Scientific	Cat# 21186

**Bacterial and virus strains**		

SARS-Related Coronavirus 2 (SARS-CoV-2) Culture Fluid (Heat Inactivated) USA-WA 1/2020	Zeptometrix	Cat# 0810587CFHI-0.5 mL
SARS-Related Coronavirus 2 (SARS-CoV-2) Lineage B.1.1.7; Alpha Variant Culture Fluid (Heat Inactivated) USA/CA*CDC*5574/2020	Zeptometrix	Cat# 0810612CFHI
SARS-Related Coronavirus 2 (SARS-CoV-2) Lineage BA.1.1.529; Omicron Variant Culture Fluid (Heat Inactivated) USA/MD-HP2087/2021	Zeptometrix	Cat# 0810642CFHI

**Chemicals, peptides, and recombinant proteins**		

Bovine Lactoperoxidase (LPO)	Sigma-Aldrich	Cat# L2005
AEBSF	Sigma-Aldrich	Cat# A8456
Aprotinin	Sigma-Aldrich	Cat# A6103
Bestatin	Sigma-Aldrich	Cat# B8385
E-64	Sigma-Aldrich	Cat# E3132
Leupeptin	Sigma-Aldrich	Cat# L2884
Pepstatin A	Sigma-Aldrich	Cat# P5318
PrimeScreen Ketone Test Strips	PrimeScreen	MPN# 754472552115
pH indicator strips (pH 4.5–10.0)	JT Baker	Cat# 4395–01
TRIzol Reagent	Invitrogen	Cat# 15596026
SuperScript IV Reverse Transcriptase	Invitrogen	Cat# 18090010

**Critical commercial assays**		

GenScript SARS-CoV-2 Neutralizing Antibody Rapid Test Kit	GenScript	Cat# DXK007
ADEXUSDx COVID-19 Test	NOWDx, Inc.	Cat#8075
COVID-19 S-Protein S1RBD Human IgG ELISA Kit	Abcam	Cat# ab274340
Amplite Universal Fluorimetric Protease Activity Assay Kit Green Fluorescence	AAT Bioquest	Cat# 13500
API ZYM system	bioMérieux	Cat# 25200
Peroxidase Activity Assay Kit	Abcam	Cat# ab155895
Lysozyme Activity Assay Kit	Abcam	Cat# ab211113
Bovine Lactoferrin ELISA Kit	Abcam	Cat# ab200015
Human Lactoferrin ELISA Kit	Novus Biologicals	Cat# NBP2-60512
2019-nCoV_N Positive Control	IDT	Cat# 10006625
TRIzol Reagent	Invitrogen	Cat# 15596026
SuperScript IV Reverse Transcriptase	Invitrogen	Cat# 18090010
ARTIC nCoV-2019 primers V4.1	IDT	N/A
plexWell 384 Library Preparation Kit	seqWell	Cat# PW384

**Deposited data**		

SARS-CoV-2 genome sequences	GISAID/NCBI	GISAID: EPI_ISL_XXXXXXXX; NCBI: XXXXXXXX (see Table S1)

**Oligonucleotides**		

ARTIC nCoV-2019 primers V4.1	IDT	N/A
2019-nCoV_N Positive Control	IDT	Cat# 10006625

**Software and algorithms**		

GraphPad Prism 7	GraphPad Software	RRID:SCR_002798
Pangolin	Rambaut et al.^49^	https://github.com/cov-lineages/pangolin
Minimap2	Li^50^	https://github.com/lh3/minimap2
iVAR	Grubaugh et al.^51^	https://github.com/andersen-lab/ivar
Samtools	Li et al.^52^	RRID:SCR_002105
Trimmomatic	Bolger et al.^53^	RRID:SCR_011848
Agilent MassHunter	Agilent Technologies	RRID:SCR_015040

**Other**		

Agilent 7900 ICP-MS	Agilent Technologies	N/A
MiSeq Sequencing System	Illumina	N/A
Leica BOND III Autostainer	Leica Biosystems	N/A
Hologic Panther System	Hologic	N/A
FilterMax F3 Multi-Mode Microplate Reader	Molecular Devices	BBBF5-1
SpectraMax ×190 Microplate Reader	Molecular Devices	2254-30-1005

### Experimental model and study participant details

#### Ethics, specimen collection, and biosafety

WTD milk, serum, and mammary gland tissue were collected from legally harvested lactating does, identified at harvest based on active milk expression and mammary gland engorgement, during the 2022–2024 hunting seasons in Amherst, Bedford, and Nelson counties, central Virginia, USA (Figure S1), after consultation with the Virginia Department of Wildlife Resources. Late-season lactation in these animals is consistent with known variation in WTD reproductive timing (38). All WTD specimens were adult females and were obtained post-mortem from animals legally harvested by licensed hunters during regulated hunting seasons on private land with landowner permission, independently of and prior to any research involvement; no animal was killed, handled, restrained, or manipulated for the purposes of this study. Accordingly, the work did not constitute animal research requiring Institutional Animal Care and Use Committee (IACUC) review under Virginia Tech policy. The study was deemed exempt under Category 2 and Category 4 by the Centra Health Institutional Review Board (CHIRB 0601e). Human milk samples from consenting lactating adult female volunteers (h.s.#1 and h.s.#2) and clinical specimens for regional variant surveillance were obtained under Virginia Tech IRB# 20-852, IRB#24-474, and Virginia Department of Health IRB# 70046, respectively, with informed consent. Human milk was obtained from two lactating female volunteers (h.s.#1 and h.s.#2; *n* = 2), aged 28 and 33 years at the time of first collection. Race, ethnicity, and ancestry were not recorded. Participants were not randomized or allocated to experimental groups; this was an observational design in which samples from each participant were stratified post hoc according to their timepoint relative to documented SARS-CoV-2 infection (pre-infection, active infection, and post-infection/late convalescence). No blinding was performed. The final sample set comprised eight lactating WTD does and two human participants. A total of eight lactating WTD does (approximately 25% of harvested does) from three counties across three hunting seasons were utilized for this study.

#### Sex as a biological variable

Only female subjects were studied. Lactation is the biological process under investigation, and milk can be obtained only from lactating females; consequently, both cohorts consist exclusively of adult females (*n* = 8 WTD does; *n* = 2 human participants). The influence of sex on the reported findings could therefore not be evaluated, and no sex-based comparisons were possible. This constraint is intrinsic to the research question rather than a consequence of cohort selection, and the findings reported here describe the properties of female mammary secretions and should not be generalized beyond that context. Gender was not a variable in this study and was not recorded for the human participants.

#### Contamination-prevention protocols for WTD milk collection

To ensure sample integrity and to prevent cross-contamination during field collection, research personnel implemented comprehensive biosafety protocols adapted for outdoor conditions. All field collection activities were conducted using personal protective equipment including disposable nitrile gloves, face masks, and protective clothing that were changed between each animal. Collection instruments were single-use and sterile, with separate sets designated for each sample type. Milk collection surfaces and teats were cleaned with 70% isopropanol prior to sampling. All samples were immediately placed in sterile, labeled containers and maintained on ice during field transport. Field collection areas were sanitized between animals using 10% bleach solution. All team members involved in sample collection were vigilant regarding any COVID-19 symptoms and provided nasal swab samples for SARS-CoV-2 testing by antigen lateral flow assay or RT-qPCR during the WTD hunting seasons. Samples were stored at -20°C for short-term and -80°C for long-term storage until analysis. Chain of custody documentation was maintained throughout.

### Method details

Milk samples were obtained from WTD does by sequentially amputating 5.0 mm of the distal teat aspect and manually expressing milk into sterile conical tubes (Figure 1B). Total milk volumes were aliquoted and stored at -20°C and -80°C until analysis. Mammary gland tissue was excised from all lactating, as well as representative non-lactating, does and fixed in 10% neutral buffered formalin for histological evaluation. Human milk samples were collected from consenting lactating volunteers at specified timepoints (for h.s.#1, 05/2023 was pre-infection and 11 months after delivery) and stored similarly.

#### SARS-CoV-2 regional variant surveillance

Nasopharyngeal swabs from suspected COVID-19 cases were submitted to Virginia Tech Molecular Diagnostics Laboratory. A total of 14,446 specimens were collected and 654 SARS-CoV-2-positive specimens (Cq ≤34) underwent whole-genome sequencing. cDNA was synthesized using SuperScript IV Reverse Transcriptase; amplification used ARTIC nCoV-2019 primers (V4.1); library preparation used the plexWell™ 384 Library Preparation Kit; sequencing was performed on MiSeq (Illumina). Bioinformatics analysis used containerized workflows including Trimmomatic trimming, Minimap2 alignment to MN908947.3 reference, Samtools processing, iVAR consensus generation, and Pangolin variant classification. Lineages were categorized as Omicron, XBB, JN.1, or other variants. All sequences were deposited in GISAID and NCBI SARS-CoV-2 repositories.

#### Histopathological analysis

Mammary gland tissues were fixed in 10% neutral buffered formalin, paraffin-embedded, sectioned at 5 μm, and stained with hematoxylin and eosin. Immunohistochemistry was performed using a mouse monoclonal antibody against SARS-CoV/SARS-CoV-2 nucleocapsid (Sino Biological, Clone 05, 1:1000) on a Leica BOND III platform (University of Virginia).

#### Detection of SARS-CoV-2 antibodies in biological fluids

Neutralizing antibodies were detected by lateral flow immunochromatography (GenScript SARS-CoV-2 Neutralizing Antibody Rapid Test Kit, which uses colloidal gold conjugates of the wild-type (Wuhan-Hu-1) spike receptor-binding domain and requires no anti-species secondary reagent; ADEXUSDx COVID-19 Test) and by quantitative ELISA (Abcam COVID-19 S-Protein S1RBD Human IgG ELISA Kit) adapted for species-independent detection using recombinant protein A/G (Thermo Scientific). Results were considered positive above 15 U/mL per manufacturer’s specifications (sensitivity 94.74%, specificity 98.57%).

#### Viral RNA detection and stability assays

Milk and nasopharyngeal swabs from WTD and human subjects were analyzed for SARS-CoV-2 by RT-qPCR targeting the *N*, *E*, and *S* genes as described.^54^ Transcription-mediated amplification (Hologic Panther System) was used for a subset of WTD samples collected in 2022. For stability assays, skim milk from WTD or human origin was spiked with ∼200,000 copies of chemically-inactivated SARS-CoV-2 variants A, B.1.1.7, or BA.1.1.529 (Zeptometrix), consisting of purified, intact viral particles that have been chemically modified to render them non-infectious while preserving particle integrity, and incubated at room temperature (∼22°C), 37°C, or 63°C for up to 60 min. Aliquots were collected in triplicate at specified timepoints, RNA extracted with TRIzol, and quantified by RT-qPCR against standard curves using 2019-nCoV_N positive controls (Integrated DNA Technologies).

#### pH and ketone body determination

Milk pH was measured using colorimetric indicator strips (JT Baker, pH 4.5–10.0). Strips were wetted with 10–20 μL milk and compared to manufacturer reference scales with results recorded to the nearest 0.5 unit. Ketone bodies were detected using PrimeScreen test strips according to manufacturer instructions, with human urine from a consenting ketonic donor used as positive control.

#### Quantitative determination of trace metals by ICP-MS

Trace metal concentrations were determined by inductively coupled plasma mass spectrometry (ICP-MS) using an Agilent 7900 instrument. Milk samples were centrifuged at 12,000×g for 10 min at 4°C, and metabolites were extracted using methanol:water (4:1 v/v). After incubation at -20°C for 30 min and re-centrifugation at 14,000×g, supernatants were dried under nitrogen and reconstituted in 50% acetonitrile with 0.1% formic acid. Data acquisition and analysis were carried out using the Agilent 7900 ICP-MS instrument (Analytical Chemistry Research Core Laboratory, Virginia Tech) with Agilent MassHunter software. Peak intensities were normalized across samples and used for downstream comparative analysis.

#### Total protease activity assay

Protease activity was measured in human and WTD milk specimens at room temperature using the Amplite® Universal Fluorimetric Protease Activity Assay Kit Green Fluorescence (AAT Bioquest), following manufacturer’s protocol A. The protease substrate was diluted 1:100 in 2x assay buffer to prepare the working solutions at pH 3, pH 7.6, and pH 8. Fluorescence intensity was measured at excitation/emission wavelengths of 490/525 nm using a microplate fluorescence reader (Molecular Devices).

#### Multi-enzyme activity profiling (API-ZYM)

Human and WTD whole milk specimens were treated at either room temperature (∼22°C) or 100°C for 5 min, then placed on ice for 15 min prior to assaying various enzymatic activities using the API® ZYM system (BioMérieux). Temperature-treated samples were diluted 1:5 and incubated at 37°C for 4 h, followed by the addition of ZYM A and ZYM B reagents for color development. After a 5 min incubation, reactions were recorded using the manufacturer’s reference reading table, with intensity scored as “-” (negative), and from weakest to strongest as “+,” “++,” “+++.”

#### Lactoperoxidase (LPO) activity assay

Human and WTD milk specimens were treated at either room temperature (∼22°C) or 100°C for 5 min, then placed on ice for 15 min prior to assaying LPO using a Peroxidase Activity Assay Kit (Abcam). Reaction mixtures consisting of skim milk, OxiRed™ probe, and H_2_O_2_ substrate were incubated at 37°C for 3 min following manufacturer’s instructions, and the initial fluorescence (Ex/Em at 535/587 nm; A_0_) was recorded using a microplate reader (Molecular Devices). After a further 60 min incubation at 37°C, final fluorescence (A_1_) was measured. Peroxidase activity was calculated from a hydrogen peroxide standard curve, using the change in fluorescence (ΔA = A_1_− A_0_), and expressed as U/µL, where one unit corresponds to the oxidation of 1.0 μmol of H_2_O_2_ per minute at 37°C.

#### Lysozyme activity assay

Lysozyme activity in skim milk was measured using the Lysozyme Activity Assay Kit (Abcam) per manufacturer's instructions. A substrate mixture combining lysozyme substrate with assay buffer was added to sample and positive control wells and incubated at 37°C for 30 min in the dark. Fluorescence was measured at Ex/Em = 360/445 nm (FilterMax™ F3 Multi-Mode Microplate Reader). Lysozyme activity (mU/mL) was calculated using a 4-methylumbelliferone (4-MU) standard curve; one unit generates 1.0 μmol 4-MU per minute at pH 5.0 and 37°C.

#### Lactoferrin concentration assay

Lactoferrin concentrations in WTD and human milk were quantified using the Bovine Lactoferrin ELISA Kit (Abcam) and the Human Lactoferrin ELISA Kit (Novus Biologicals), respectively, following manufacturers' instructions. Skim milk was treated at room temperature or 100°C as described above. Absorbance was measured at 450 nm (Molecular Devices SpectraMax X190), and lactoferrin concentrations were interpolated from standard curves and adjusted for dilution.

#### Protease inhibition and ionic supplementation assays

Human skim milk (h.s.#1, 05/2023) was supplemented with an ion cocktail mimicking WTD milk mineral concentrations (Na^+^ 20.9 mM, Mg^2+^ 2 mM, P 8.6 mM, K^+^ 20.8 mM, Ca^2+^ 0.74 mM, Fe^3+^ 6.7 μM, Zn^2+^ 11 μM), incubated on ice for 20 min, and treated with or without a protease inhibitor cocktail (AEBSF 1 mM, aprotinin 800 nM, bestatin 50 μM, E-64 15 μM, leupeptin 20 μM, and pepstatin A 10 μM) prior to virus spiking (∼200,000 viral RNA copies). A parallel protocol included an intermediate heat-shock step (100°C, 5 min) followed by 15 min on ice. WTD milk (L-D4) was tested under protease inhibition alone, given its endogenous high ionic concentrations. For reconstitution assays, human milk was supplemented with ions and/or LPO (0.4 U/μL, Sigma) to assess individual and combined antiviral effects.

### Quantification and statistical analysis

All experiments were performed in triplicate unless otherwise noted. Data are presented as mean ± SD. Differences between two groups were analyzed by Student's t-test; differences among more than two groups by one-way ANOVA with Holm–Sidak's multiple comparisons test or by multiple t test comparison (GraphPad Prism 7). *p* ≤ 0.05 was considered statistically significant. Viral copy numbers were quantified by RT-qPCR against standard curves using 2019-nCoV_N positive controls (IDT). Statistical significance is indicated in all figures as: ∗*p* <0.05, ∗∗*p* <0.01, ∗∗∗*p* <0.001, ∗∗∗∗*p* <0.0001; n.s., not significant.

## Supplemental information

Document S1. Figures S1–S3

Table S1. GISAID and NCBI accession numbers for the 654 SARS-CoV2 genome sequence during regional variant surveillance in southwest Virginia, 2022–2024
