# Supplementary material for: White-tailed deer milk promotes rapid SARS-CoV-2 RNA degradation through synergistic ionic and enzymatic mechanisms
Source: iScience. 2026 Nov 6;29(11):118136. doi: 10.1016/j.isci.2026.118136 (PMC13760985; doi:10.1016/j.isci.2026.118136)
Supplement: Supplementary file 1 [file mmc1.pdf]

iScience, Volume *29*

Supplemental information

White-tailed deer milk promotes rapid SARS-CoV-2 RNA degradation through synergistic ionic and enzymatic mechanisms

Lorena P. Tobar, Wraylyn Smith, Hannah A. Raso, Katherine L. Brown, Alessandro Ceci, Matthew G. Urbano, Clinton Roby, Dzenis Mahmutovic, Andrew P. Biesemier, Daniel G.S. Capelluto, Ignacio Aiello, Dominic S. Raso, and Carla V. Finkielstein

A.

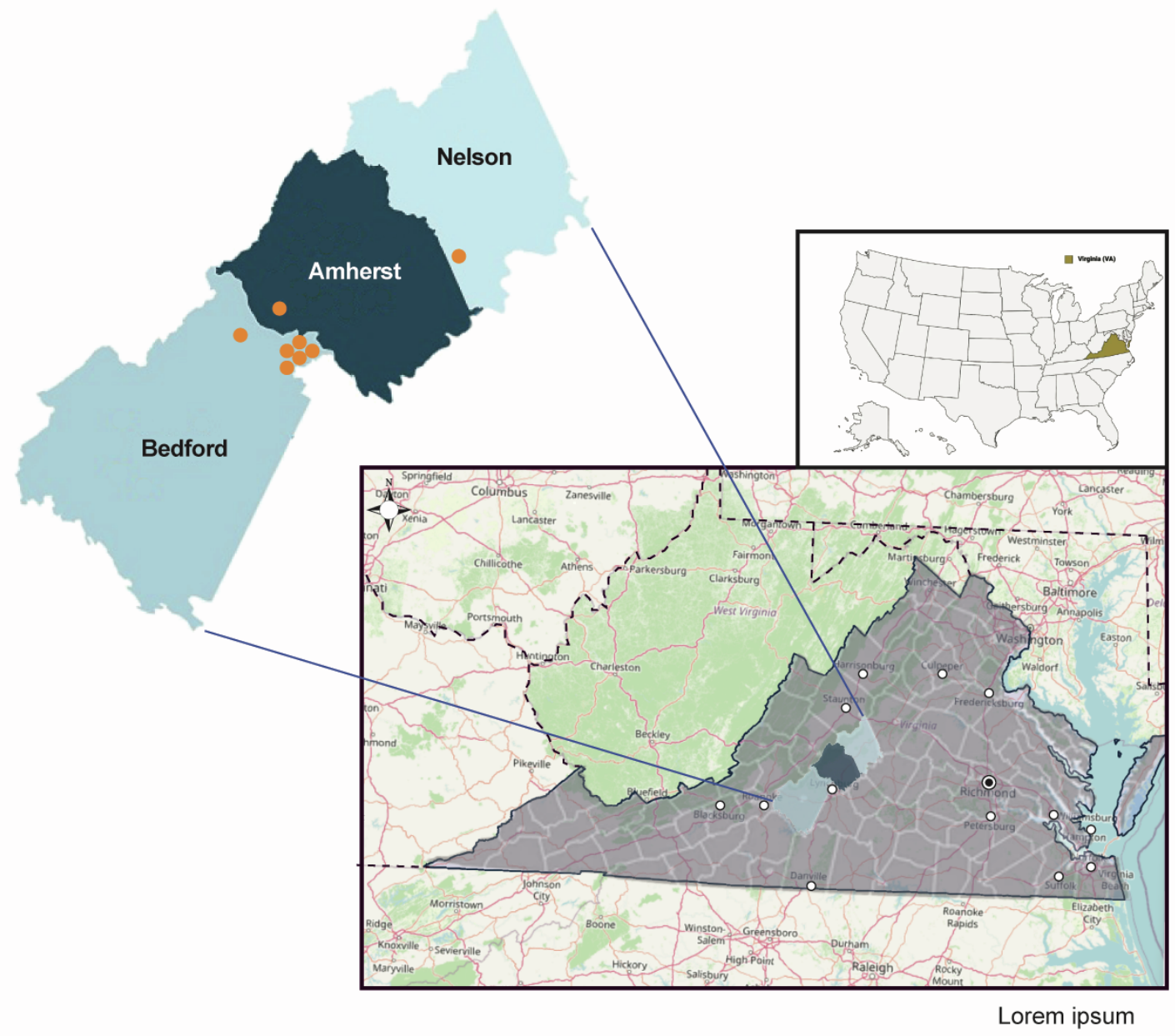

2022-2024 Central Virginia WTD harvest				
County	Hectares	WTDs	Hectares/WTD	% Female
Amherst	122,759	3,084	40	41.6
Bedford	196,872	7,451	27	50.1
Nelson	121,928	1,987	62	30.0

B.

WTD tested	Collection date	County	Biochem./Molec.assay
A04	11/11/22	Bedford	lateral flow/ELISA/RTPCR/NGS/Ketone/pH/API-ZYM/other enz. assay
A07	10/09/22	Bedford	lateral flow/ELISA/RTPCR/NGS/API-ZYM/other enz. assay
A08	10/09/22	Bedford	lateral flow/ELISA/RTPCR/NGS/Mass Spec./Ketone/prot. activity/pH/API-ZYM/other enz. assay
A09	10/27/22	Bedford	lateral flow/ELISA/RTPCR/NGS/Ketone/prot. activity/API-ZYM/other enz. assay
A14	11/21/22	Amherst	lateral flow/ELISA/RTPCR/NGS/Ketone/prot. activity/pH/API-ZYM/other enz. assay
A23	11/26/22	Bedford	lateral flow/ELISA/RTPCR/NGS/prot. activity/API-ZYM/other enz. assay
LD4	11/04/23	Nelson	lateral flow/ELISA/RTPCR/NGS/Mass Spec./Ketone/prot. activity/pH/API-ZYM/other enz. assay
LD20	10/28/24	Bedford	lateral flow/RTPCR/NGS/API-ZYM/other enz. assay

Figure S1. Geographic distribution and hunting context of WTD specimen collection in central Virginia, USA. (A) Lactating white-tailed deer were sampled from three adjacent central Virginia counties that differ in reported deer density and in the female fraction of the regulated harvest. WTD harvest occurred on private land with permission in central Virginia during the 2022-2024 hunting seasons in Amherst, Bedford, and Nelson counties. Participating licensed hunters preferentially targeted does (approximately 66% of total harvested WTD) at landowner request, resulting in 8 lactating does (approximately 25% of harvested does) utilized for this study. The map shows county boundaries with color-coding corresponding to sample density. Individual sampling locations are marked with colored dots: orange dots indicate female deer. The lactational WTD county distribution table (lower right) details the geographic harvest locations of the 8 lactating does. The inset map (left) shows the geographic location within Virginia, USA. The 2022-2024 central Virginia WTD harvest table (upper right) provides county-specific information including total area (hectares) and three-year estimates (mean) for total number, density, and percent female of reported WTD harvest based on Virginia Department of Wildlife Resources (VDWR) data. Note the relatively high WTD density, especially in Bedford County and to a lesser extent in Amherst and Nelson counties. This hunting approach reflects VDWR management goals to stabilize county-specific populations through liberalized regulated hunting, especially for females, to minimize impacts on herd health, property, and human safety (http://www.virginiawildlife.gov/wildlife/deer/management-plan). (B) Eight lactating does constitute the specimen set analyzed in this study, with assay coverage differing among individual animals.

Summary of individual WTD specimens, listing animal identifier, county and date of harvest, and the assays performed on each sample.

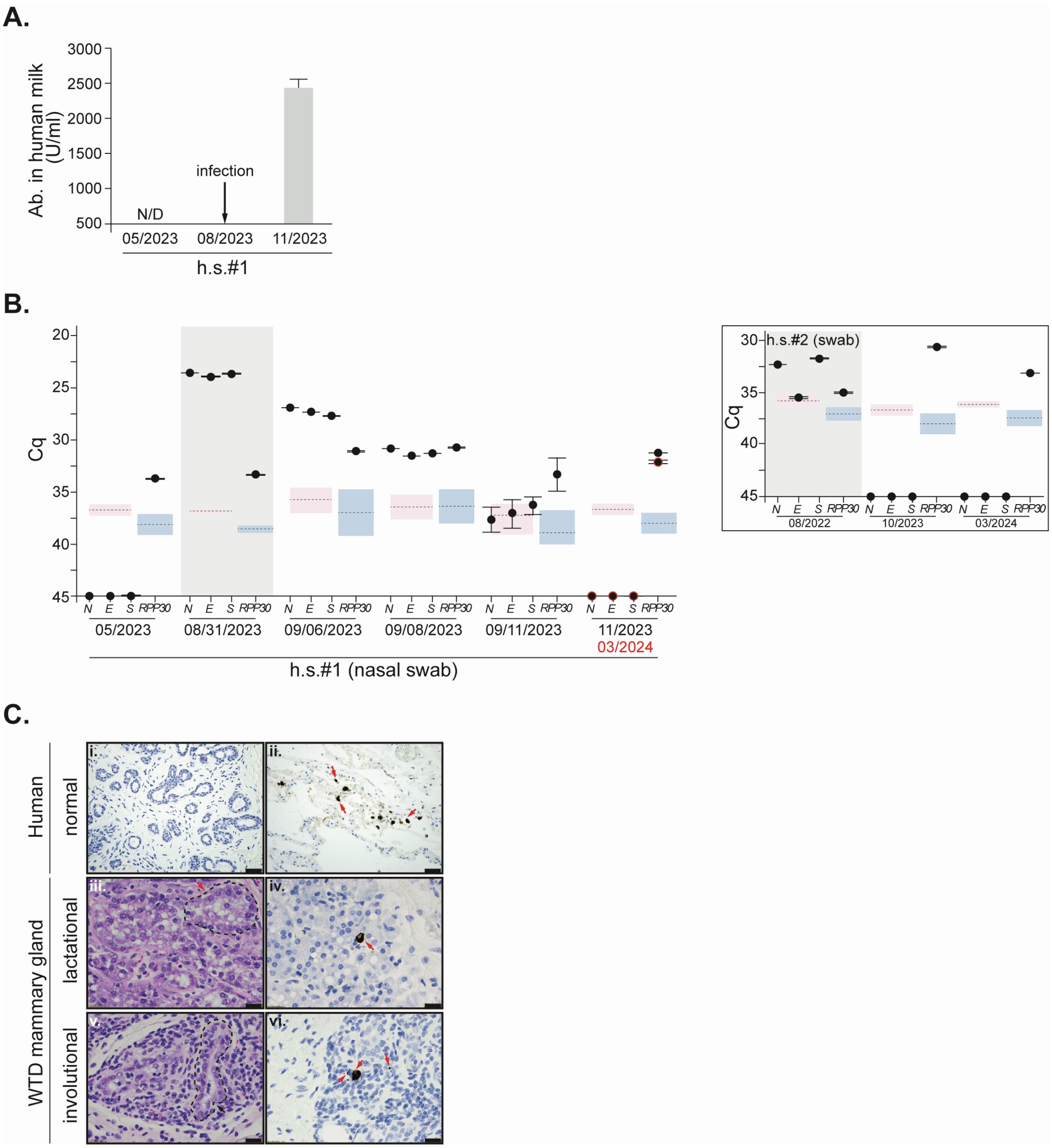

Figure S2. Viral RNA detection, human milk antibody kinetics, and histopathological analysis. (A) Antibody reactivity in milk from h.s.#1 was not detected before infection and was measurable at late convalescence. Antibody levels in human milk from h.s.#1 measured by ELISA at three timepoints: pre-infection (05/2023, N/D), post-infection (08/2023), and late convalescence (11/2023). Arrow indicates timing of documented infection. Error bars indicate SD. (B) Nasal swab RT-qPCR follows the appearance and subsequent loss of detectable SARS-CoV-2 RNA across the sampled timepoints in both human

subjects. RT-qPCR analysis of human nasal swabs showing SARS-CoV-2 RNA detection (N, E, S genes) and human RPP30 control. Cq values are plotted for h.s.#1 at multiple timepoints pre- and post-infection. Inset shows RT-qPCR results for h.s.#2 at the time of infection. Plotted values indicate mean ± SD of a triplicate. Detection thresholds and corresponding SD are indicated as dashed lines with pink and blue shades for viral and RPP30 genes, respectively. Cq values below the threshold indicate a positive result (note the y-axis is inverted). Gray shading indicates an active infection period. (C) Mammary gland architecture and SARS-CoV-2 nucleocapsid immunoreactivity are compared between human and WTD tissue. Normal mammary gland histological examination (SARS-CoV-2 negative) (i.) and post-mortem SARS-CoV-2 nucleocapsid-positive immunohistochemistry in lung (ii., arrows). Hematoxylin and eosin (H&E)-stained mammary gland lobules from lactating (iii.) and non-lactating involutional (v.) WTD does. Note coalescing alveoli composed of vacuolated secretory cells within the lactating lobule and inactive alveoli with intervening stroma and lymphocytes within the involutional lobule (alveolus - dashed outline). Rare SARS-CoV-2-positive histiocytes (arrows) are present within lactating (iv.) and non-lactating (vi.) mammary gland lobules. H&E, original magnification 40× (iii., v.); SARS-CoV-2 immunohistochemistry original magnification, 20× (i., ii.) and 40× (iv., vi.). Images captured with Canon Eos 70D (i., ii.) and Olympus DP 72 (iii., iv., v., vi.) cameras. Scale bars represent 50 µm.

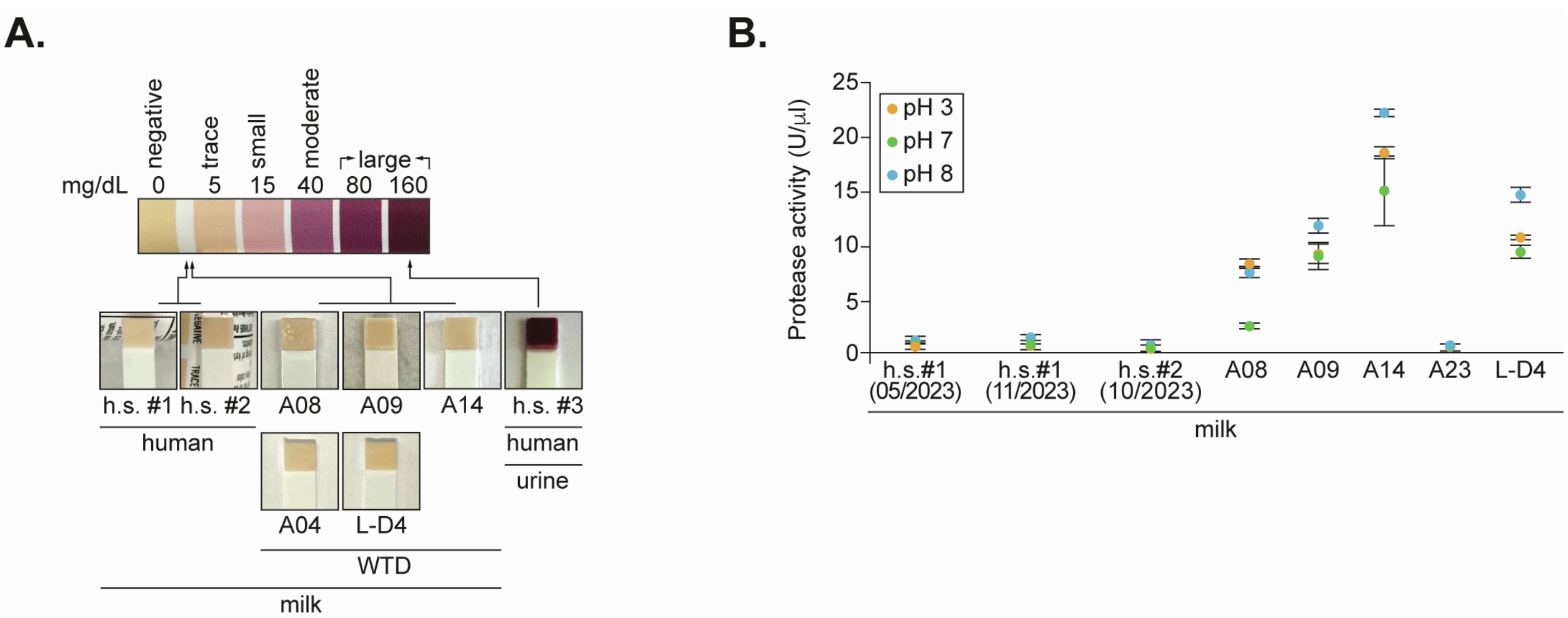

Figure S3. Ketone body and protease activity measurements. (A) Ketone reactivity in WTD milk remained at the low end of the test strip scale relative to a ketone-positive human urine control. Ketone body detection in milk samples using PrimeScreen test strips. Color intensity correlates with ketone concentration (from 0 to >160 mg/dL scale). Human milk (h.s.#1, h.s.#2) and WTD milk (A08, A09, A04, L-D4, A14) are shown. A human urine sample (h.s.#3) from a known ketone-positive donor is included as a positive control. (B) Protease activity varied among WTD milk samples. Protease activity measurements in human (h.s.#1, h.s.#2) and WTD milk samples (A08, A09, A14, A23, and L-D4) at pH 3, 7, and 8 using fluorometric assay. Activity values are reported as corrected fluorescence units (U/µl). Error bars represent mean ± SD from triplicate measurements.
